# Thromboembolic Events During Treatment with Cisplatin-based Chemotherapy in Metastatic Testicular Germ-cell Cancer 2000–2014: A Population-based Cohort Study

**DOI:** 10.1016/j.euros.2021.07.007

**Published:** 2021-08-15

**Authors:** Hege S. Haugnes, Helene F. Negaard, Hilde Jensvoll, Tom Wilsgaard, Torgrim Tandstad, Arne Solberg

**Affiliations:** a Department of Oncology, University Hospital of North Norway, Tromsø, Norway; b Department of Clinical Medicine, UiT The Arctic University, Tromsø, Norway; c Department of Oncology, Oslo University Hospital, Oslo, Norway; d Department of Hematology, University Hospital of North Norway, Tromsø, Norway; e Department of Community Medicine, UiT The Arctic University, Tromsø, Norway; f The Cancer Clinic, St. Olavs Hospital, Trondheim, Norway; g Department of Clinical and Molecular Medicine, The Norwegian University of Science and Technology, Trondheim, Norway

**Keywords:** Arterial thromboembolism, Cisplatin, Testicular cancer, Venous thromboembolism, Thromboprophylaxis, Bleeding

## Abstract

**Background:**

Cisplatin-based chemotherapy (CBCT) in testicular cancer (TC) is associated with elevated venous thromboembolism (VTE) risk, but trials evaluating the safety and efficacy of thromboprophylaxis are lacking.

**Objective:**

To evaluate the arterial thromboembolism (ATE) and VTE incidence and risk factors during first-line CBCT for metastatic TC, and the effect of thromboprophylaxis on VTE and bleeding.

**Design, setting, and participants:**

In a population-based study, 506 men administered first-line CBCT during 2000–2014 at three university hospitals in Norway were included. Clinical variables were retrieved from medical records.

**Outcome measurements and statistical analysis:**

Patients with ATE and VTE diagnosed at initiation of or during CBCT until 3 mo after completion were registered. Age-adjusted logistic regression was performed to identify possible VTE risk factors.

**Results and limitations:**

Overall, 69 men (13.6%) were diagnosed with 70 thromboembolic events. Twelve men (2.4%) experienced ATE. Overall, 58 men (11.5%) experienced VTE, of whom 13 (2.6%) were prevalent at CBCT initiation, while 45 (8.9%) were diagnosed with incident VTE. Age-adjusted logistic regression identified retroperitoneal lymph node metastasis >5 cm (odds ratio [OR] 1.99, 95% confidence interval [CI] 1.01–3.91), central venous access (OR 2.84, 95% CI 1.46–5.50), and elevated C-reactive protein (>5 mg/l; OR 2.38, 95% CI 1.12–5.07) as incident VTE risk factors. Thromboprophylaxis (*n* = 84) did not influence the risk of VTE (VTE incidence with or without prophylaxis 13% vs 8%, *p* = 0.16). The incidence of bleeding events was significantly higher among those who received thromboprophylaxis than among those without thromboprophylaxis (14.5% vs 1.1%, *p* < 0.001).

**Conclusions:**

We found a high rate of thromboembolism incidence of 13.6%. Thromboprophylaxis did not decrease the risk of VTE but was associated with an increased risk of bleeding.

**Patient summary:**

We found a high rate of thromboembolism (13.6%) during cisplatin-based chemotherapy for metastatic testicular cancer. Prophylactic treatment against thromboses did not reduce the thrombosis frequency, but it resulted in a high incidence of bleeding events.

## Take Home Message

We found a high rate of thromboembolism (13.6%) during cisplatin-based chemotherapy for metastatic testicular cancer. Prevalent venous thromboembolism (VTE) was more frequent in men with retroperitoneal lymph nodes >5 cm, intermediate/poor prognosis disease, poor performance status, and elevated C-reactive protein. Consequently, we advise to examine these patients closely with regard to VTE.

Central venous access and large retroperitoneal lymph node metastases were associated with an increased risk of incident VTE in age-adjusted analysis. Patients without any risk factors had 5% incidence of VTE, indicating a thrombotic potential of cisplatin-based chemotherapy. Prophylactic treatment against thromboses did not reduce the thrombosis frequency, but it resulted in a high incidence of bleeding events.

## 1 Introduction

Testicular cancer (TC) treatment is a medical success story, with 10-yar overall survival approaching 90% even in metastatic disease [1]. The excellent prognosis in advanced TC was primarily achieved by the introduction of cisplatin in the late 1970s and with standardized diagnostics, treatment, and follow-up [2]. However, the general health of these young patients may be impaired by treatment-related morbidity, including thromboembolism.

Cancer patients have a four- to seven-fold higher risk of venous thromboembolism (VTE) than the general population [3]. VTE is among the leading causes of noncancer mortality among cancer patients [4]. The life-threatening potential of thromboembolism in TC patients was demonstrated by two recent large studies, reporting five- to seven-fold increased risks of death from cardiovascular disease (CVD), including VTE, during the 1st year after cisplatin-based chemotherapy (CBCT) [5], [6].

Incidence rates of arterial thromboembolism (ATE) between 0.3% and 1.2% during CBCT for metastatic TC have been reported previously [7], [8], [9]. However, separate ATE risk factors in this population are evaluated incompletely. In recent studies, the incidence of VTE during CBCT for TC ranges from 9% to 19% [8], [9], [10], [11], [12], [13], [14]. The most important risk factors identified were International Germ Cell Cancer Collaborative Group (IGCCCG) intermediate and poor prognosis groups [15], large retroperitoneal lymph node (RPLN) metastases, and central venous access. Importantly, no randomized trials have evaluated the safety and efficacy of thromboprophylaxis in metastatic TC.

The aims of this population-based cohort study were to evaluate ATE and VTE incidence and risk factors during primary CBCT for metastatic TC. Furthermore, we aimed to evaluate the effect of thromboprophylaxis, incidence of bleeding complications, and impact of thromboembolism on overall survival.

## 2 Patients and methods

### 2.1 Patients

Treatment of metastatic germ-cell TC is centralized to four university hospitals in Norway, with treatment and follow-up according to the Swedish and Norwegian Testicular Cancer Group (SWENOTECA) protocols [16]. The study patients were prospectively registered in local SWENOTECA databases and comprise all Norwegian men who initiated primary CBCT for metastatic germ-cell TC at two of the four university hospitals during 2000–2014 and at one hospital during 2008–2014. Men with primary metastatic disease and first relapse after initial stage I disease were included. This study was approved by the Regional Ethical Committee for Medical Research Ethics (REK 2015/602).

Chemotherapy consisted of three cycles of cisplatin, etoposide, and bleomycin (BEP) or four cycles of cisplatin plus etoposide for IGCCCG good prognosis patients. Intermediate and poor prognosis patients received four cycles of BEP. Primary chemotherapy was intensified in case of poor tumor marker decline with the addition of ifosfamide (first step) and, for some, high-dose chemotherapy as the second step [17]. Granulocyte colony stimulating factor and antiemetic medications were used according to international guidelines.

### 2.2 Variables

Clinical variables and details regarding thromboembolism diagnosis and treatment were retrieved from medical records. Disease and treatment variables included diagnosis date, histology, clinical stage (Royal Marsden staging system) [18], size and location of metastases, IGCCCG prognosis group [15], use and type of central venous access, and treatment details. Clinical variables registered at the start of CBCT included performance status, height and weight (to calculate body mass index [BMI]; kg/m^2^), medication, smoking status, comorbidity, and standard laboratory analyses (tumor markers, hemoglobin, leukocyte count, platelet count, C-reactive protein [CRP], and creatinine). Cause and date of death were registered.

Thromboembolic events were defined according to international clinical practice as objectively confirmed ATE (myocardial infarction [MI], ischemic stroke, and other arterial events) or VTE (pulmonary embolism and deep vein thrombosis [proximal or distal]). Events were diagnosed shortly before or at the initiation of CBCT (prevalent events), or during CBCT until 3 mo after completion (incident events). Diagnostic criteria for MI included clinical symptoms, electrocardiogram findings, and elevated cardiac enzymes. Other ATE and VTE events were confirmed radiographically (computed tomography [CT] scan and ultrasound), including symptomatic VTE (imaging performed on suspicion of VTE) and incidental VTE (imaging performed for other reasons, eg, cancer staging or treatment evaluation).

Thromboprophylaxis was not the standard treatment during the study period and was given at the discretion of the treating physician. The use and type of thromboprophylaxis were registered. Only patients who received thromboprophylaxis for a minimum of 7 d were categorized as receiving such treatment [11]. Bleeding events throughout the study period were registered and classified as fatal, major (bleeding at a critical site and/or requiring transfusions with minimum two units of red cells and/or a fall in hemoglobin level of 2 g/dl) [19], or minor (clinically relevant nonmajor events).

The longest axial diameter of RPLN metastasis was registered, and dichotomized with a 5-cm cutoff [10]. Khorana score was calculated based on the presence of TC and cutoff levels for BMI, hemoglobin, leukocyte, and thrombocyte count [20]. Creatinine clearance was estimated based on serum creatinine and age [21], with 90 ml/min/1.73 m^2^ as the cutoff for normal kidney function [22]. Elevated CRP was defined as a value of >5 mg/l (upper normal limit).

### 2.3 Statistical analysis

Continuous variables are presented as median (interquartile range [IQR]), and categorical variables are presented as counts (proportion). Groups were compared using the chi-square test. The overall observation time (in years) was calculated from the date of first CBCT cycle until death or the end of follow-up (as of May 2020). Days to first thromboembolic event was calculated from the date of first CBCT cycle until thromboembolism occurred.

Analyses of possible risk factors for incident VTE were performed after the exclusion of 13 patients with prevalent VTE at the start of CBCT, since only incident events can be prevented. Age-adjusted and multivariable logistic regression was performed, presented with odds ratios (ORs) and 95% confidence intervals (CIs). In a multivariable regression analysis, significant variables from age-adjusted analyses were included using the backward Wald selection (forward selection gave similar results).

Cumulative survival was calculated with the Kaplan-Meier method. The association between any thromboembolic events during treatment and overall mortality was assessed using age-adjusted Cox regression, presented as hazard ratio (HR) and 95% CI. Statistical analyses were performed using the SPSS 26.0 package (SPSS Inc., Chicago, IL, USA). Two-sided *p* values of <0.05 were considered significant.

## 3 Results

### 3.1 Patient characteristics

**Fig. 1:**
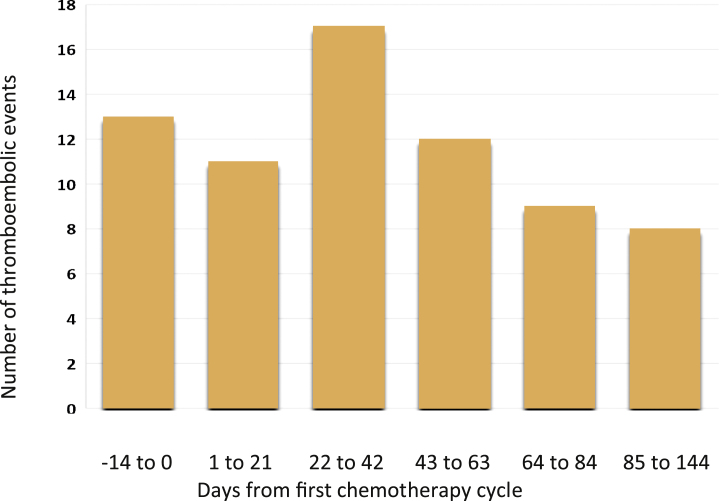
A histogram showing the number of thromboembolic events according to days from the initiation of the first chemotherapy cycle, grouped according to the duration of each chemotherapy cycle until the end of cycle 4. Each cycle lasts for 21 d.

In total, 506 patients were included (Supplementary Fig. 1). The median age at CBCT initiation was 33.4 yr (IQR 18–48), and the median observation time was 8.7 yr (IQR 1.9–15.4; Table 1). The majority had nonseminoma (62%) and belonged to the IGCCCG good prognosis group (81%). Before or during treatment, 70 thromboembolic events occurred in 69 men (13.6%; Fig. 1). One had both MI and pulmonary embolism (Table 2).

**Table 1:** Disease and treatment characteristics for 506 germ-cell testicular cancer patients treated with first-line cisplatin-based chemotherapy for metastatic disease during 2000–2014

Characteristic	Overall
Institution	
St Olavs University Hospital	207 (41)
Oslo University Hospital, Ullevaal	188 (37)
University Hospital of North Norway	111 (22)
Indication for cisplatin-based chemotherapy	
Primary metastatic disease	400 (79)
Relapse treatment a	106 (21)
Age at chemotherapy initiation (yr), median (IQR)	33.4 (18–48)
Observation time (yr), median (IQR)	8.7 (1.9–15.4)
Histology	
Seminoma	194 (38)
Nonseminoma	312 (62)
Stage at time of chemotherapy (Royal Marsden)	
I Mk+	22 (4)
II	340 (67)
III	35 (7)
IV	109 (22)
Size of retroperitoneal metastases	
No retroperitoneal metastases	48 (10)
IIA (<2 cm)	113 (22)
IIB (2–5 cm)	237 (47)
IIC (>5 cm)	108 (21)
Tumor markers at diagnosis, median (IQR)	
HCG (IU/l)	4.9 (0–58.8)
AFP (μg/l)	4.0 (0–27.5)
LD (U/l)	209 (33–385)
Patients with elevated markers at diagnosis	
HCG	239 (47)
AFP	175 (35)
LD	229 (45)
Prognostic group b	
Good prognosis	412 (81)
Intermediate prognosis	54 (11)
Poor prognosis	40 (8)
Chemotherapy type, first regimen	
BEP	368 (73)
EP	117 (23)
PEI	21 (4)
Treatment intensification	
None	456 (90)
PEI/BEP-IF only	35 (7)
PEI/BEP-IF followed by high dose	11 (2)
PEI/BEP-IF followed by TIP	4 (1)
Type of venous access	
Peripheral venous access	415 (82)
Central venous catheter^c^	79 (16)
Venous port	12 (2)

AFP = alpha-fetoprotein; BEP = bleomycin, etoposide, cisplatin; BEP-IF = bleomycin, etoposide, cisplatin, ifosfamide; EP = etoposide, cisplatin; HCG = human chorionic gonadotropin; IQR = interquartile range; LD = lactate dehydrogenase; Mk+ = marker positive; PEI = cisplatin, etoposide, ifosfamide; PICC = peripherally inserted central catheter; TIP = paclitaxel, ifosfamide, cisplatin.

Data are presented as *n* (%) unless otherwise specified. There are missing data for some of the variables (HCG, *n* = 1; AFP, *n* = 1; LD, *n* = 29).

a Of 106 patients, 104 had stage I disease initially, of whom 92 relapsed while under surveillance. Two patients relapsed after radiotherapy for initially stage IIA disease.

b According to the International Germ Cell Cancer Collaboration Group [15].

c None of which were PICC line.

**Table 2:** Type and location of thromboembolic event (TE) according to chemotherapy timing among 506 germ-cell testicular cancer patients treated with first-line cisplatin-based chemotherapy for metastatic disease during 2000–2014

Type of TE	Total		Prevalent TE	Incident TE	
				During chemo	After chemo
Arterial embolism					
In total		12 (2.4)	0	11 (2.2)	1 (0.2)
Myocardial infarction a		5 (1.0)	0	5	0
Cerebral infarction		2 (0.4)	0	2	0
Kidney infarction		1 (0.2)	0	0	1
Occlusion of limb arteries b		4 (0.8)	0	4	0
Venous thromboembolism					
In total	58 (11.5)		13 (2.6)	35 (6.9)	10 (2.0)
Pulmonary embolism a	30 (5.9)		2	21	7
Abdominal DVT	10 (2.0)		7	1	3
Lower limb DVT	10 (2.0)		3	6	0
Upper limb DVT	6 (1.2)		0	6	0
Other c	2 (0.4)		1	1	0

chemo = chemotherapy; DVT = deep vein thrombosis.

Data are presented as *n* (%).

a One patient with myocardial infarction also had pulmonary embolism 5 d after the end of chemotherapy, while still on platelet inhibition.

b One a. poplitea, one a. iliaca comm, one a. femoralis, and one a. brachilalis.

c One internal jugular vein and one superior caval vein.

### 3.2 ATE incidence and risk factors

Overall, 12 men had ATE (2.4%). The majority (*n* = 11) occurred during chemotherapy (Table 2). The median time from CBCT initiation to ATE diagnosis was 37 d (IQR 24–48). Five events were MI (1% of the total study population). Overall, 11 ATE patients were symptomatic. One was asymptomatic, identified at CT evaluation (renal infarction). There were no ATE-related deaths.

The median age at CBCT initiation for patients with ATE was 51 yr (IQR 40–53), considerably higher than for those without thromboembolic events (median age 32.4 yr, *p* < 0.001). Whereas 92% of patients diagnosed with ATE had IGCCCG good prognosis disease, 11 of 12 men had one or more CVD risk factors, most commonly smoking (*n* = 8) or obesity (*n* = 5). One patient had pre-existing CVD (stroke; Supplementary Table 1).

### 3.3 VTE incidence and risk factors

Overall, 58 men (11.5%) had VTE (Table 2). Thirteen men (2.6%) were prevalent VTE events at TC diagnosis, while 45 men (8.9%) were incident VTE event at a median of 46 d (IQR 3–89) after the initiation of CBCT. Pulmonary embolism was the most common VTE (*n* = 30). There was one VTE-related death (pulmonary embolism).

The median age at CBCT initiation in 13 men with prevalent VTE was 46 yr (IQR 32–60; Table 3). Eight men (62%) had symptomatic VTE. The majority of patients with prevalent VTE had RPLN >5 cm (92%), IGCCCG intermediate/poor prognosis disease (54%), poor performance status (62%), and elevated CRP (92%).

**Table 3:** Possible risk factors for VTE among all included men (*N* = 506) overall and according to VTE status and timing

Characteristic	Overall (*N* = 506)	Without VTE (*N* = 448)	Prevalent VTE (*N* = 13)	Incident VTE (*N* = 45)
Age at chemotherapy initiation (yr), median (IQR)	33.4 (18–48)	32.4 (17–47)	46.0 (32–60)	35.9 (23–49)
RPLN axial diameter (cm) a				
≤5	398 (79)	366 (82)	1 (8)	31 (69)
>5	108 (21)	82 (18)	12 (92)	14 (31)
Prognostic group b				
Good	412 (81)	372 (83)	6 (46)	34 (76)
Intermediate	54 (11)	45 (10)	4 (31)	5 (11)
Poor	40 (8)	31 (7)	3 (23)	6 (13)
Patients with markers above normal				
HCG	239 (47)	205 (46)	8 (62)	26 (58)
AFP	175 (35)	152 (34)	6 (46)	17 (38)
LD	230 (45)	192 (43)	13 (100)	25 (56)
Patients with abnormal hematology				
Hemoglobin <10 g/dl	8 (1.6)	5 (1.1)	3 (23)	0
Leukocyte count >11 × 10^9^/l	35 (7)	26 (5.8)	5 (39)	4 (8.9)
Platelets ≥350 × 10^9^/l	71 (14)	55 (12.3)	7 (54)	9 (20)
Obesity (BMI ≥30 kg/m^2^)	80 (16)	69 (15)	2 (15)	9 (20)
Khorana score c				
1	347 (67)	316 (70)	1 (23)	28 (62)
2	87 (17)	71 (16)	5 (39)	11 (24)
≥3	24 (4.7)	17 (4)	5 (39)	2 (4)
Current smoker	155 (31)	139 (31)	3 (23)	13 (29)
Central venous access	91 (18)	72 (16)	3 (23)	16 (36)
Thromboprophylaxis ≥7 d d	84 (17)	73 (16)	NA	11 (24) e
Past history with VTE or coagulopathy	1	0	0	1
Immobilization	12 (2.3)	6 (1.3)	0	6 (13)
Performance status				
ECOG 0	360 (71)	326 (73)	4 (31)	30 (67)
ECOG ≥1	57 (11)	44 (10)	8 (62)	5 (11)
Creatinine clearance ≤90 ml/min/1.73 m²	83 (16)	62 (14)	9 (70)	12 (27)
CRP >5 mg/l	138 (27)	110 (25)	12 (92)	16 (36)

AFP = alpha-fetoprotein; BMI = body mass index; CRP = C-reactive protein; ECOG = Eastern Cooperative Oncology Group; HCG = human chorionic gonadotropin; IQR = interquartile range; LD = lactate dehydrogenase; *N* = numbers; RPLN = retroperitoneal lymph node; VTE = venous thromboembolic events.

Data are presented as *n* (%) unless otherwise specified. All data based on laboratory and clinical examinations are at initiation of first chemotherapy cycle. There are missing data for some of the variables: HCG, *n* = 1; AFP, *n* = 1; LD, *n* = 29; hemoglobin, *n* = 35; leukocyte count, *n* = 43; platelets, *n* = 47; obesity, *n* = 1; Khorana score, *n* = 48; current smoker, *n* = 28, ECOG status, *n* = 89; creatinine clearance, *n* = 44; CRP, *n* = 129.

a Only the 5 cm cutoff was associated with VTE risk [10]. The 3.5 cm cutoff was not significantly associated with VTE risk and is not reported [11].

b According to the International Germ Cell Cancer Collaborative Group [15].

c Khorana score was calculated based on the presence of testicular cancer, and cut-off levels for BMI, hemoglobin, leukocyte and thrombocyte count [20].

d Among 84 men with thromboprophylaxis, 81 men had low-molecular weight heparin (LMWH; *n* = 81), of whom 77 had low-dose LMWH (ie, enoxaparin 40 mg daily or dalteparin 5000 E daily) and four had LMWH in therapeutic dosage as prophylaxis (ie, enoxaparin 120 mg daily). Three received platelet inhibitors, for example, acetylsalicylic acid 160 mg daily.

e Nine men were diagnosed with VTE while still on thromboprophylaxis and one after termination of thromboprophylaxis, and one had unknown disease.

The median age of the 45 men diagnosed with incident VTE was 36 yr at CBCT initiation (Table 3), and 31 of them (69%) had symptomatic VTE. Of these men, 14 (31%) had RPLN >5 cm, 16 (36%) had central venous access, and 16 (36%) had elevated CRP.

In age-adjusted logistic regression analyses, RPLN >5 cm, central venous access, and elevated CRP (>5 mg/l) were significantly associated with the risk of incident VTE (Table 4). A Khorana score of ≥3 was not associated with VTE risk. In the multivariable logistic regression analysis, only central venous access (OR 2.70, 95% CI 1.18–6.19) were significantly associated with VTE.

**Table 4:** Possible risk factors for incident VTE among 493 men at risk

Variable	Age-adjusted analysis		Multivariable analysis	
	OR	95% CI	OR	95% CI
Age at diagnosis, per year	1.02	0.99–1.05		
RPLN metastasis diameter (cm) a				
≤5	Reference			
>5	1.99	1.01–3.91		
Prognostic group b				
Good	Reference			
Intermediate	1.30	0.48–3.50		
Poor	2.29	0.88–5.93		
Lactate dehydrogenase				
Within normal range	Reference			
Above upper limit	1.77	0.93–3.37		
Khorana score c				
1	Reference			
2	1.73	0.82–3.64		
≥3	1.33	0.29–6.08		
Central venous access				
No	Reference		Reference	
Yes	2.84	1.46–5.50	2.70	1.18–6.19
Performance status				
ECOG 0	Reference			
ECOG ≥1	1.20	0.44–3.27		
Creatinine clearance				
>90 ml/min/1.73 m^2^	Reference			
≤90 ml/min/1.73 m^2^	2.02	0.93–4.39		
CRP at diagnosis, dichotomized				
≤5 mg/l	Reference		Reference	
>5 mg/l	2.38	1.12–5.07	1.93	0.88–4.23

BMI = body mass index; CI = confidence interval; CRP = C-reactive protein; ECOG = Eastern Cooperative Oncology Group; OR = odds ratio; RPLN = retroperitoneal lymph node; VTE = venous thromboembolic event.

Age-adjusted and multivariable logistic regression. Overall, 13 men with prevalent VTE at initiation of chemotherapy were excluded. There are missing data for some of the variables: Khorana score, *n* = 48; performance status, *n* = 88; creatinine clearance, *n* = 44; CRP, *n* = 129.

a Only the 5 cm cutoff was associated with VTE risk [10]. The 3.5 cm cutoff was not significantly associated with VTE risk and is not reported [11].

b According to the International Germ Cell Cancer Collaborative Group [15].

c Khorana score was calculated based on the presence of testicular cancer, and cutoff levels for BMI, hemoglobin, leukocyte, and thrombocyte count [20].

Overall, 196 men had none of the significant VTE risk factors identified in age-adjusted logistic regression models. Their incidence of VTE during chemotherapy was 4.6%, as compared with 13% among men with a minimum of one risk factor (*p* = 0.003).

### 3.4 Thromboprophylaxis and VTE

Overall, 84 patients (17%) received thromboprophylaxis with a median duration of 89 d (IQR 35–143). Only four men received thromboprophylaxis for <25 d, and no patients received thromboprophylaxis for <7 d. The majority of patients (*n* = 81) received low-molecular-weight heparin (LMWH), of whom 77 received low-dose LMWH (Table 3).

Overall, 11 of 84 men (13%) given thromboprophylaxis were diagnosed with incident VTE, as compared with 34 of 409 men (8%) among those without thromboprophylaxis (*p* = 0.16). VTE risk factors were more frequent among those who received thromboprophylaxis (RPLN >5 cm 42% vs 14%; poor prognosis disease 21% vs 5%; central venous access 34% vs 14%; CRP >5 mg/l 42% vs 22%). However, among men with a minimum of one of the three significant VTE risk factors identified in age-adjusted logistic regression models, thromboprophylaxis did not reduce VTE incidence (15% with prophylaxis vs 14% without prophylaxis, *p* = 0.83).

### 3.5 Bleeding complications

The incidence of bleeding events in the study population was 4.2% (*n* = 21; Table 5). Overall, seven bleeding events occurred after the initiation of full-dose anticoagulation (10%). The incidence of bleeding events was significantly higher among those who received thromboprophylaxis (14%) than among those without thromboprophylaxis (1.1%; *p* < 0.001).

**Table 5:** Patients with bleeding events according to anticoagulation status

Bleeding event	Total (*N* = 506)	Full-dose anticoagulation (*N* = 69)	On thromboprophylaxis (*N* = 70)	Without thromboprophylaxis (*N* = 367)
Any bleeding event	21 (4.2)	7 (10)	10 (14)	4 (1.1)
Fatal bleeding event	1 (0.2)	1 (1.5)	0	0
Major bleeding event	5 (1.0)	2 (2.9)	2 (2.9)	1 (0.3)
In brain metastases	2		2	
Muscle hematoma	1	1		
Bladder	1	1		
Severe nose bleed	1			1
Minor bleeding event	15 (3.0)	4 (5.8)	8 (11)	3 (0.8)
Nose bleed	7	1	5	1
Hemoptysis	2	1		1
Hemorrhoid	2	1	1	
Hematuria	2		1	1
Central venous access	2	1	1	

*N* = numbers.

Data are presented as *n* (%). Bleeding events are classified as fatal, major (cerebral bleeding or requiring surgery or transfusions), or minor. Germ-cell testicular cancer patients treated with first-line cisplatin-based chemotherapy for metastatic disease during. 2000-2014.

Bleeding was fatal (bleeding after RPLN dissection while on anticoagulation for pulmonary embolism) in one patient and major (none related to surgery) in five patients, of whom two (2.9%) were on full-dose anticoagulation, two (2.9%) were on thromboprophylaxis, and one (0.3%) was without thromboprophylaxis. Most bleeding events (*n* = 15) were minor.

### 3.6 Mortality

Overall, 37 patients died during follow-up (7.3%). The median time from CBCT initiation to death was 1.8 yr (range 0.01–13.8 yr). Causes of death were germ-cell TC (*n* = 18), treatment related (*n* = 5), CVD (*n* = 5), second malignant neoplasm (*n* = 1), and other causes (*n* = 8). The cumulative 10-yr overall survival was 94% (95% CI 92–96) among men without thromboembolism and 87% (95% CI 79–95) after any thromboembolic event.

In age-adjusted Cox regression, we observed a borderline significant association between prevalent or incident thromboembolism and overall mortality (HR 1.98, 95% CI 0.94–4.19). However, when including the prognosis group in the model, the association disappeared (HR 1.18, 95% CI 0.53–2.61).

## 4 Discussion

In this population-based cohort study, we found a thromboembolism incidence of 13.6% during primary CBCT for metastatic TC. Risk factors for incident VTE included RPLN >5 cm, central venous access, and elevated CRP. Importantly, thromboprophylaxis was not associated with a reduction in the VTE incidence, but with a high incidence of bleeding events, mostly minor.

Our reported incidence rates of ATE (2.4%) and MI (1%) are considerably higher than the 0.3–1.2% ATE and 0.2–0.4% MI incidence rates reported previously [7], [8], [9]. In line with previous literature [23], men with ATE were older than those without thromboembolic events, and the majority had a minimum of one CVD risk factor. Still, these 12 men were considerably younger than the Norwegian general population at MI diagnosis (median age 51 vs 69 yr) [24], suggesting that CBCT-induced acute endothelial dysfunction might cause ATE [25].

The 11.5% VTE incidence rate confirms data from previous large studies [10], [11], [12]. In total, 2.6% of our patients had prevalent VTE, corroborating data from a large Spanish study [12] but lower than the rates of 4.4–6.5% reported by others [10], [11]. Risk factors for prevalent VTE at TC diagnosis have not been reported previously. We found that prevalent VTE was more frequent in men with RPLN >5 cm, intermediate/poor prognosis disease, poor performance status, and elevated CRP. Consequently, we advise to examine these patients closely with regard to symptoms and/or radiologic findings, raising a suspicion of VTE.

Overall, 8.9% of our study patients were diagnosed with an incident VTE, supporting results from two large studies [10], [11]. In line with previous large studies [8], [9], [10], [11] and a recent literature review [26], we found that central venous access and large RPLN metastases were associated with an increased risk of incident VTE in age-adjusted analysis. Patients without any risk factors had 5% incidence of VTE, indicating a thrombotic potential of CBCT. We did not identify a high Khorana score as a risk factor for incident VTE, in contrast to two previous studies [10], [11]. However, a high Khorana score (≥3) was present in 39% of patients with prevalent VTE at TC diagnosis, probably reflecting advanced metastatic TC.

Elevated CRP at CBCT initiation was associated with an increased risk of incident VTE in our study, suggesting a proinflammatory state, rendering these men susceptible for the thrombotic potential of CBCT. To our knowledge, this is a novel finding in the TC patient population. Inflammation is important in the VTE pathogenesis in general [27], [28] and among cancer patients [29]. A previous study among TC patients found elevated white blood cells to be associated with VTE [10], also reflecting the possible impact of inflammation.

Recent randomized trials evaluating direct oral anticoagulants (DOACs) as thromboprophylaxis in ambulatory cancer patients given chemotherapy reported 60% risk reductions for VTE, with a double risk of bleeding [30], [31]. Although the American Society of Clinical Oncology clinical practice guideline recommends thromboprophylaxis with DOACs or LMWH to selected high-risk ambulatory patients [32], no data from randomized trials support the routine use in TC patients, as the fraction of TC patients in recent trials was very small (<1%). Owing to the paucity of randomized data, a recent European Association of Urology guideline recommends balancing each patient’s benefits and risk of thromboprophylaxis [33]. In line with previous reports [9], [11], thromboprophylaxis did not reduce the incidence of VTE in our study, possibly due to the selection of patients with VTE risk factors for thromboprophylaxis. Although not statistically significant, Gizzi et al [8] reported 45% less VTE with thromboprophylaxis versus no thromboprophylaxis in a study among 151 TC patients with VTE risk factors (nine/97 vs nine/54, *p* = 0.23). However, a study reporting a 19% VTE incidence among 255 TC patients, of whom 93% received LMWH thromboprophylaxis, failed to show any effect of thromboprophylaxis [9].

Regarding thromboprophylaxis, the risk of bleeding complications must be taken into consideration. As many as 14% of our patients on thromboprophylaxis experienced a bleeding event. Even though these events were predominately minor, the proportion was considerably higher than among men without thromboprophylaxis (14% vs 1.1%, *p* < 0.001). In addition, the overall incidence of bleeding among men with thromboprophylaxis was considerably higher than the 2.5% reported in the Global Germ Cell Cancer Group (G3) study [34]. The 2.9% major bleeding incidence with thromboprophylaxis, mainly with LMWH, in our study was in line with the 2–3.5% reported in randomized trials using DOACs [30], [31].

According to previous reports, cancer patients who develop thromboembolism, in particular VTE, have increased mortality during follow-up [3]. While some previous studies confirmed the adverse prognosis among TC patients with thromboembolism [9], [12], neither our results nor the G3 study [11] confirmed this association when adjusting for the IGCCCG prognosis group.

Strengths of this relatively large study include the population-based design, homogeneous clinical practice across participating centers, and a predefined study population including only men administered first-line CBCT for metastatic TC. Data were extracted from medical records with a low risk of misclassification bias and a high likelihood of completeness. Limitations include missing data for some laboratory variables and skewness regarding selection of a low number of patients for thromboprophylaxis. To adjust for risk factors, a nonrandomized evaluation of thromboprophylaxis should ideally include a larger cohort than reported so far [8], [9], [11]. In our opinion, analyses on thromboprophylaxis reflecting clinical routine in this relatively large cohort is still important, given the absence of data from randomized trials and the rarity of metastatic TC.

## 5 Conclusions

In conclusion, although CBCT has a high thrombogenic potential, as demonstrated by the 5% incidence among men without any VTE risk factors, our study does not support the routine use of low-dose LMWH to prevent VTE. Given the high incidence of bleeding and the fact that VTE in this patient population did not influence survival, thromboprophylaxis should be considered only in selected patients. The most important risk factor for incident VTE seems to be central venous access use, which should be avoided in routine clinical practice [33].

  ***Author contributions*:** Hege Sagstuen Haugnes had full access to all the data in the study and takes responsibility for the integrity of the data and the accuracy of the data analysis.

  *Study concept and design*: Haugnes, Negaard, Tandstad, Solberg.

*Acquisition of data*: Haugnes, Negaard, Solberg.

*Analysis and interpretation of data*: Haugnes, Negaard, Jensvoll, Wilsgaard, Tandstad, Solberg.

*Drafting of the manuscript*: Haugnes, Negaard, Jensvoll, Wilsgaard, Tandstad, Solberg.

*Critical revision of the manuscript for important intellectual content*: Haugnes, Negaard, Jensvoll, Wilsgaard, Tandstad, Solberg.

*Statistical analysis*: Haugnes, Wilsgaard.

*Obtaining funding*: None.

*Administrative, technical, or material support*: Haugnes.

*Supervision*: None.

*Other*: None.

  ***Financial disclosures:*** Hege Sagstuen Haugnes certifies that all conflicts of interest, including specific financial interests and relationships and affiliations relevant to the subject matter or materials discussed in the manuscript (eg, employment/affiliation, grants or funding, consultancies, honoraria, stock ownership or options, expert testimony, royalties, or patents filed, received, or pending), are the following: None.

  ***Funding/Support and role of the sponsor***: None.
