# Supplementary material for: Thromboembolic Events During Treatment with Cisplatin-based Chemotherapy in Metastatic Testicular Germ-cell Cancer 2000–2014: A Population-based Cohort Study
Source: Eur Urol Open Sci. 2021 Aug 15;32:19–27. doi: 10.1016/j.euros.2021.07.007 (PMC8505199; doi:10.1016/j.euros.2021.07.007)
Supplement: Supplementary file 1 [file mmc1.docx]

**Supplementary Figure 1. Flow chart for study patients and different outcomes.**

Germ-cell testicular cancer patients treated with first-line cisplatin-based chemotherapy for metastatic disease during 2000-2014 (n=506) (Table 1, Table 3)

Exclusion of men with VTE at chemotherapy start (n=13)

Overall 69 men (13.6%) with 70 TE (Table 2, Figure)

- 13 men with prevalent events (before start of chemotherapy, only VTE)
- 56 men with incident events during/after chemotherapy (45 VTE, 12 ATE of whom one with ATE and VTE)

Overall 493 men available for calculation of risk factors for incident VTE (n=45) (Table 4) and for thromboprophylaxis calculations:

- 11/84 (13%) men with prophylaxis had VTE
- 34/409 (8%) without prophylaxis had any VTE

All 506 men available for estimation of bleeding complications (Table 5)

Effect of thromboprophylaxis on bleeding complications analyzed in 437 men without TE (Table 5)

- 70 men (13.8%) on thromboprophylaxis only
- 367 men (72.5%) without any thromboprophylaxis or full-dose anticoagulation

Abbreviations: TE, thromboembolic events; VTE, venous thromboembolic events; ATE, arterial embolic events.
