# Supplementary material for: Thromboembolic Events During Treatment with Cisplatin-based Chemotherapy in Metastatic Testicular Germ-cell Cancer 2000–2014: A Population-based Cohort Study
Source: Eur Urol Open Sci. 2021 Aug 15;32:19–27. doi: 10.1016/j.euros.2021.07.007 (PMC8505199; doi:10.1016/j.euros.2021.07.007)
Supplement: Supplementary file 2 [file mmc2.docx]

Supplementary Table 1 - Cardiovascular risk factors among 12 patients with arterial events during/after cisplatin-based chemotherapy.

| Patient no. | Current smoking | | | Obesity | Hypertension | Hyper-cholesterolemia | Diabetes | No. of risk factors | | Pre-existing CVD |
| --- | --- | --- | --- | --- | --- | --- | --- | --- | --- | --- |
| 1 | | Yes | Yes | | No | No | No | | 2 | No |
| 2 | | Yes | | Yes | No | No | No | | 2 | No |
| 3 | | Yes | | No | No | No | No | | 1 | No |
| 4 | | Yes | | No | No | No | No | | 1 | No |
| 5 | | Yes | | No | No | No | No | | 1 | No |
| 6 | | Yes | | No | No | No | No | | 1 | No |
| 7 | | No | | No | No | No | No | | 0 | No |
| 8 | | No | | Yes | No | No | No | | 1 | No |
| 9 | | No | | No | Yes | No | No | | 1 | No |
| 10 | | Yes | | No | No | No | No | | 1 | No |
| 11 | | Yes | | Yes | No | No | No | | 2 | Yes |
| 12 | | No | | Yes | Yes | Yes | Yes | | 4 | No |
| TOTAL | | 8 | | 5 | 2 | 1 | 1 | |  | 1 |

Abbreviations: No; numbers; CVD, cardiovascular disease
